# Clinical manifestations and outcomes of HSK following corneal transplantation

**DOI:** 10.3389/fmed.2025.1654643

**Published:** 2025-08-20

**Authors:** Dan Wu, Hui Huang, Min Zheng, Qi Chen, Zhou Zhou, Li Jiang, Yuanhua Li, Yiyun Liu, Baikai Ma, Guoqing Chen, Yujun Huang, Fengping Cen, Binghua Chen, Fengmei Li, Hong Qi, Fan Xu, Qianqian Lan

**Affiliations:** 1 Department of Ophthalmology, The People’s Hospital of Guangxi Zhuang Autonomous Region & Guangxi Key Laboratory of Eye Health & Guangxi Health Commission Key Laboratory of Ophthalmology and Related Systemic Diseases Artificial Intelligence Screening Technology & Institute of Ophthalmic Diseases, Guangxi Academy of Medical Sciences, Nanning, China; 2 Department of Ophthalmology, Peking University Third Hospital & Beijing key Laboratory of Restoration of Damaged Ocular Nerve, Peking University Third Hospital, Beijing, China

**Keywords:** herpes simplex keratitis, corneal transplantation, new-onset HSK, recurrent HSK, HSK classification

## Abstract

**Purpose:**

To investigate the clinical manifestations and outcomes of herpes simplex keratitis (HSK) infection following corneal transplantation.

**Methods:**

This retrospective study analyzed medical records of patients who underwent corneal transplantation at the People’s Hospital of Guangxi Zhuang Autonomous Region between January 2018 and March 2024, with a minimum follow-up period of 1 year. The study examined post-transplantation herpes simplex virus (HSV) infections, including the timing of HSV infection, HSK classification, clinical manifestations, and outcomes.

**Results:**

A total of 411 patients (corresponding to 411 eyes) were followed up. Among them, 88 cases (21.41% of the 411 cases) were diagnosed with or suspected of HSK before corneal transplantation. Of these 88 cases, 13 cases (14.77% of the 88 cases) developed recurrent HSK after surgery (8 cases underwent corneal transplantation during the acute phase, and 5 cases during the scarring phase). There were 323 cases with no evidence of HSK before corneal transplantation, among which 27 cases (8.36% of the 323 cases) developed new-onset HSK after corneal transplantation. Among all 411 patients, a total of 40 cases (9.73% of the 411 cases) developed HSK after corneal transplantation, with 26 cases (26 eyes) (65% of the 40 cases) developing HSK within 1–3 months post-surgery. Regarding HSK types, epithelial HSK occurred in 11 cases (27.5% of the 40 cases), all of which achieved corneal transparency with regular use of antiviral medication. Stromal necrotizing HSK occurred in 8 cases (20% of the 40 cases), with 2 cases developing corneal nebula, 5 cases developing corneal macula, and 1 case requiring repeat corneal transplantation due to near-perforation. Combined epithelial, stromal, and endothelial HSK occurred in 17 cases (42.5% of the 40 cases), with 1 case developing corneal nebula, 4 cases developing corneal macula, 10 cases developing corneal leucoma, and 2 cases requiring repeat corneal transplantation due to corneal perforation. Endothelial HSK occurred in 4 cases (10% of the 40 cases), with 2 cases achieving grade 0 corneal transparency and 2 cases achieving grade 2 corneal transparency.

**Conclusion:**

The first 1–3 months following corneal transplantation is a peak time for HSK. Regular follow-up is essential for all patients post-surgery. For those with a history of HSK, vigilance for HSK is critical, while timely diagnosis and differentiation of HSK are crucial for non-HSK patients. When administering systemic antiviral therapy, it is important to adjust the frequency and intensity of steroid treatments promptly to prevent irreversible graft opacity.

## 1 Introduction

Globally, infectious keratitis is the fifth leading cause of blindness (1), with corneal diseases ranking second among blinding eye conditions in China (2). For many individuals suffering from blindness worldwide, corneal transplantation is the only viable option for vision restoration. The prevalence of herpes simplex virus type 1 (HSV-1) is notably high, with a lifetime exposure rate of at least 60% across various populations. This figure is even higher in developing countries, reaching nearly 100% in traditional societies. Recent studies highlight that herpes simplex keratitis (HSK) following corneal transplantation is a significant contributor to graft failure. Notably, research by Remeijer et al. (3) found that the incidence of clinically diagnosed herpetic keratitis after penetrating keratoplasty (PKP) is more than 14 times higher compared to non-transplant populations. For ophthalmologists, accurately diagnosing post-transplant HSK, recognizing its clinical features, and selecting appropriate treatments present significant challenges. Many practitioners may not fully understand the clinical manifestations of HSK following transplantation, which can lead to misdiagnosis and inappropriate treatment, adversely affecting patient vision and graft clarity. Therefore, it is essential to master the clinical characteristics, treatment options, and outcomes associated with HSK after corneal transplantation.

## 2 Materials and methods

### 2.1 Study subjects

This retrospective study analyzed patients who underwent corneal transplantation at the People’s Hospital of Guangxi Zhuang Autonomous Region between January 2018 and March 2024, with a minimum follow-up period of 1 year postoperatively. The study examined postoperative HSK, including the timing of HSV infection, HSK classification, clinical manifestations, and outcomes. All the study participants were informed in advance about the study in a language they were familiar with, and written consent was taken prior to the collection of samples. The study was conducted in accordance with the Declaration of Helsinki, and approved by the Guangxi Zhuang Autonomous Region People’s Hospital (Approval Number: No. KY-ZC-2016-623) and was performed in accordance with the Declaration of Helsinki.

### 2.2 Diagnostic criteria

The diagnostic criteria for postoperative HSK following corneal transplantation included: (1) Etiological diagnosis: HSK can be definitively diagnosed when HSV testing is positive in samples such as diseased corneal tissue or aqueous humor. (2) Clinical diagnosis meeting any of the following criteria: ① Epithelial HSK: Fluorescein sodium staining of corneal lesions demonstrating typical dendritic or geographic keratitis patterns of the corneal epithelium; ② Endothelial HSK: Presence of endothelial deposits with corresponding stromal and epithelial edema, accompanied by iritis, where stromal edema may present in disciform, diffuse, or linear patterns (4, 5); ③ Corneal *in vivo* confocal microscopy: Findings consistent with HSK morphology, with exclusion of fungal and Acanthamoeba infections. All criteria mentioned in ①–③ demonstrated effective response to systemic antiviral monotherapy. Patients meeting criterion ① or criterion ②, with clinical exclusion of concurrent infections with other pathogens, were diagnosed with postoperative HSK following corneal transplantation.

### 2.3 Treatment protocol

All patients were individually selected for medications and frequency based on their comprehensive systemic condition, and treatment was continued until the condition stabilized, followed by dose reduction. (1) Nucleoside drugs: The systemic antiviral treatment regimen was as follows: treatment dose was acyclovir 200mg each time, 5 times daily, or valacyclovir 300–500 mg each time, 2 times daily; maintenance dose was acyclovir 400 mg daily. For HSK patients undergoing corneal transplantation during acute infection phase, treatment dose was given perioperatively, and after clinical assessment of stable condition, it was changed to maintenance dose for 3–6 months postoperatively; for primary HSK or recurrent HSK after corneal transplantation, treatment dose was immediately given, and after clinical assessment of stable condition, it was changed to maintenance dose; for HSK patients in corneal scar stage undergoing surgical treatment, maintenance dose was given perioperatively and continued for 2 months postoperatively (7). (2) Corticosteroid drugs: ① Tobramycin dexamethasone eye drops, 2–4 times daily. ② Tobramycin dexamethasone eye ointment, 1–2 times daily.

### 2.4 Outcome measures

#### 2.4.1 New-onset HSK and recurrent HSK

1. New-onset HSK: First clinical diagnosis of HSK following corneal transplantation in patients with no pre-operative clinical manifestations, signs, or etiological evidence of HSV infection.
2. Recurrent HSK: HSK history was confirmed or highly suspected before corneal transplantation; for patients with acute infection phase HSK undergoing corneal transplantation, HSK lesions reappeared after effective control of HSV infection; for corneal scar stage HSK, HSK lesions were first discovered postoperatively.

#### 2.4.2 Clinical classification of postoperative HSK

1. Epithelial HSK: The graft remains nearly transparent with dendritic or geographic corneal epithelial defects. Following resolution, the cornea may regain transparency or retain residual corneal nebulae.
2. Necrotizing stromal HSK: Characterized by dense corneal stromal infiltration accompanied by ulceration and necrosis. In severe cases, rapid corneal thinning and even perforation may occur within a short period.
3. Endothelial HSK: Presents with varying degrees and extent of corneal edema accompanied by Descemet’s membrane folds, without stromal infiltration or neovascularization. Gray-white keratic precipitates (KPs) are visible on the endothelial surface, corresponding to the edematous area, becoming more prominent after edema resolution.

#### 2.4.3 Outcome assessment criteria

1. Transparency grading for non-endothelial HSK:
  Transparent graft: The graft is clear with distinct visualization of iris details.
  Corneal nebula: Mild opacity presenting as a gray-white semi-transparent appearance, thin like a cloud, with iris texture still visible through the opaque area.
  Corneal macula: Moderately thick opacity with gray-white coloration, through which the iris remains faintly visible, representing an intermediate stage between nebula and leucoma.
  Corneal leucoma: Most severe opacity presenting as milk-white or porcelain-white appearance with complete obscuration of iris visualization.
  Graft perforation: Full-thickness corneal defect with partial or complete prolapse of aqueous humor or intraocular contents.
2. Transparency grading for endothelial HSK: Scored on a scale from 0 to 3 as follows:
  Grade 0: Clear graft with distinct visualization of iris details.
  Grade 1: Partially obscured iris details.
  Grade 2: Iris details indiscernible with only the pupillary margin visible.
  Grade 3: Complete obscuration of iris and pupillary details.

## 3 Results

### 3.1 Baseline characteristics

This study followed a total of 411 patients (411 eyes), including 243 males (243 eyes) (59.12% of the 411 cases) and 168 females (168 eyes) (40.88% of the 411 cases). The oldest patient was 84 years old, the youngest was 1 year old, with a mean age of 47.73 years. Among all patients, 323 cases (323 eyes) (78.59% of the 411 cases) underwent penetrating keratoplasty, 57 cases (57 eyes) (13.87% of the 411 cases) underwent deep anterior lamellar keratoplasty, and 31 cases (31 eyes) (7.54% of the 411 cases) underwent endothelial keratoplasty.

HSK after corneal transplantation occurred in 40 cases (40 eyes) (9.73% of the 411 corneal transplant patients), including 24 males (24 eyes) (60% of the 40 cases) and 16 females (16 eyes) (40% of the 40 cases). The oldest patient was 71 years old, the youngest was 24 years old, with a mean age of 47.5 years. Among these cases, 35 cases (35 eyes) (87.5% of the 40 cases) underwent penetrating keratoplasty, 3 cases (3 eyes) (7.5% of the 40 cases) underwent deep anterior lamellar keratoplasty, and 2 cases (2 eyes) (5% of the 40 cases) underwent endothelial keratoplasty.

### 3.2 Primary diseases

Among the 411 patients (411 eyes), there were 12 cases (12 eyes) (2.92% of the 411 cases) of corneal burns, 8 cases (8 eyes) (1.95% of the 411 cases) of corneal dystrophy, 13 cases (13 eyes) (3.16% of the 411 cases) of immune-related keratopathy, 51 cases (51 eyes) (12.41% of the 411 cases) of keratoconus, 17 cases (17 eyes) (4.14% of the 411 cases) of corneal dermoid, 65 cases (65 eyes) (15.82% of the 411 cases) of acute fungal keratitis, 24 cases (24 eyes) (5.84% of the 411 cases) of acute bacterial keratitis, 7 cases (7 eyes) (1.7% of the 411 cases) of other viral keratitis with etiological confirmation, 17 cases (17 eyes) (4.14% of the 411 cases) of other etiological corneal diseases, 33 cases (33 eyes) (8.03% of the 411 cases) of etiologically confirmed herpes simplex viral keratitis, and 164 cases (164 eyes) (39.9% of the 411 cases) of corneal leucoma [among which 55 cases (55 eyes) were highly suspected of previous HSK based on medical history and clinical signs, while 109 cases (109 eyes) had no evidence of HSK preoperatively]. Therefore, among the 411 patients (411 eyes), a total of 88 cases (88 eyes) (21.41% of the 411 cases) had HSK before corneal transplantation, while 323 cases (323 eyes) (78.59% of 411 the cases) had no evidence of HSK.

Among the 40 cases (40 eyes) of patients with HSK after corneal transplantation, 13 cases (13 eyes) (32.5% of the 40 cases) were recurrent HSK (14.77% of the 88 cases with HSK before corneal transplantation). Among these, 8 cases (8 eyes) underwent corneal transplantation during the acute phase of HSK, and 5 cases (5 eyes) underwent corneal transplantation during the scarring phase.

**Figure 1:**
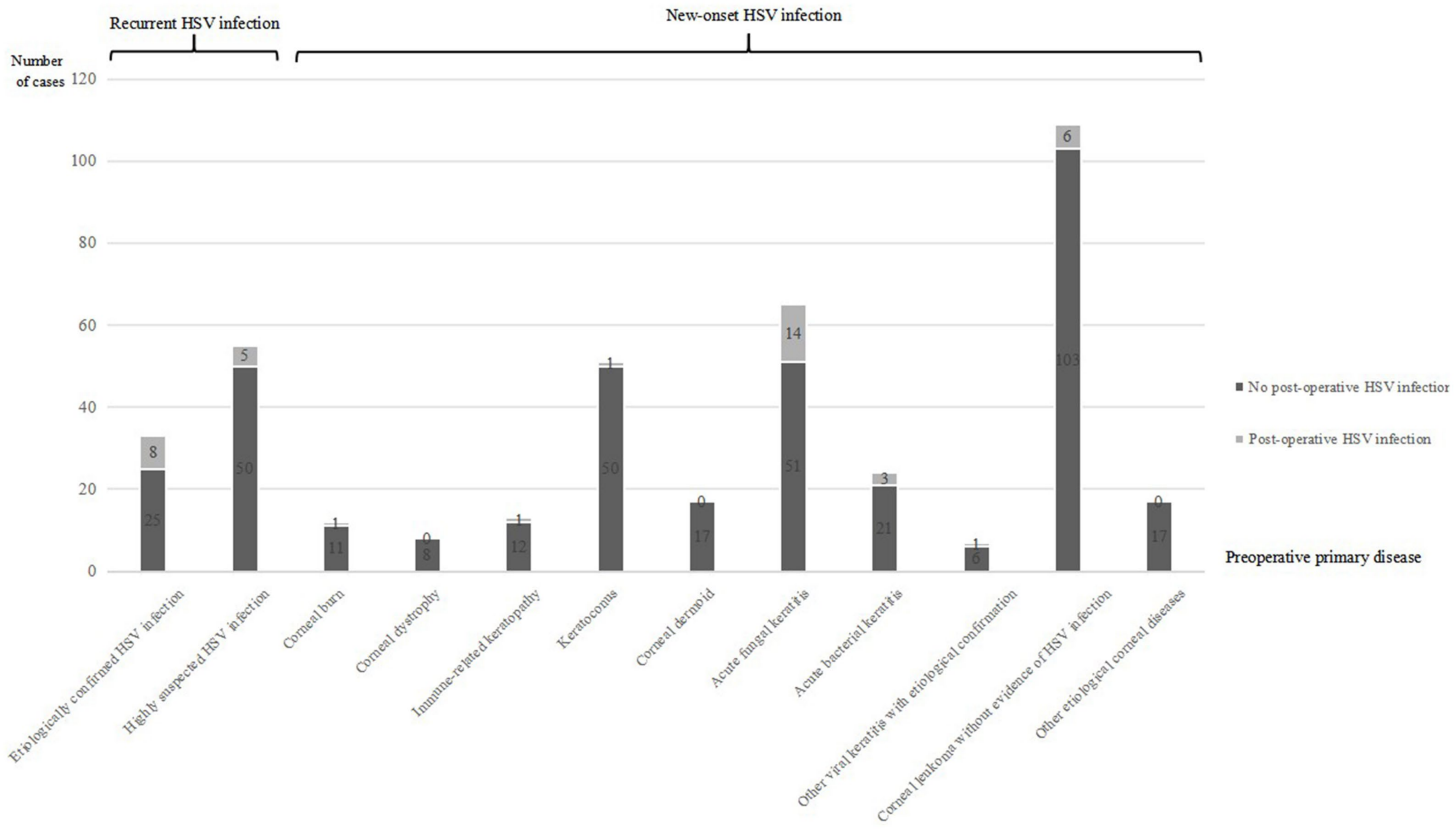
This bar chart demonstrates the number of postoperative HSK cases among total follow-up patients categorized by different preoperative disease types. Categories include etiologically confirmed HSV infection, highly suspected HSV infection, corneal burns, corneal dystrophy, immune-related keratopathy, keratoconus, corneal dermoid, acute fungal keratitis, acute bacterial keratitis, other viral keratitis with etiological confirmation, corneal leukoma without evidence of HSV infection, and other etiological corneal diseases. Each category is subdivided by presence or absence of postoperative HSV infection.

Twenty-seven cases (27 eyes) (67.5% of the 40 cases) were new-onset HSK after corneal transplantation (8.36% of the 323 cases with no evidence of HSK before corneal transplantation). The primary diseases included: 1 case (1 eye) (2.5% of the 40 cases) of corneal burn, 1 case (1 eye) (2.5% of the 40 cases) of keratoconus, 1 case (1 eye) (2.5% of the 40 cases) of immune-related keratitis, 3 cases (3 eyes) (7.5% of the 40 cases) of acute bacterial keratitis, 14 cases (14 eyes) (35% of the 40 cases) of acute fungal keratitis, 1 case (1 eye) (2.5% of the 40 cases) of etiologically confirmed viral keratitis caused by other viruses, and 6 cases (6 eyes) (15% of the 40 cases) of corneal leucoma with no evidence of HSK (Figure 1).

### 3.3 Timing of postoperative HSK

Among the 40 cases (40 eyes) with HSK after corneal transplantation, the timing of HSV infection was as follows: 26 cases (26 eyes) (65% of the 40 cases) within 1–3 months, 4 cases (4 eyes) (10% of the 40 cases) within 3–6 months, 5 cases (5 eyes) (12.5% of the 40 cases) within 6–12 months, and 5 cases (5 eyes) (12.5% of the 40 cases) beyond 12 months.

Among the 13 cases (13 eyes) with recurrent HSK after corneal transplantation, the timing of HSV infection was as follows: 8 cases (8 eyes) (61.54% of the 13 cases) within 1–3 months, 2 cases (2 eyes) (15.38% of the 13 cases) within 3–6 months, and 3 cases (3 eyes) (23.08% of the 13 cases) within 6–12 months. Among 8 cases (8 eyes) of HSK patients in the acute infection phase undergoing corneal transplantation, the time for HSV infection control was 2.1 ± 1.9 months postoperatively, and the time of HSK recurrence after discontinuation of antiviral drugs was 7.6 ± 2.2 months postoperatively. Among 5 cases (5 eyes) of HSK patients in corneal scar stage undergoing corneal transplantation, HSK occurred at 3.9 ± 2.73 months postoperatively.

Among the 27 cases (27 eyes) with new-onset HSK after corneal transplantation, the timing of HSK was as follows: 18 cases (18 eyes) (66.67% of the 27 cases) within 1–3 months, 2 cases (2 eyes) (7.41% of the 27 cases) within 3–6 months, 2 cases (2 eyes) (7.41% of the 27 cases) within 6–12 months, and 5 cases (5 eyes) (18.52% of the 27 cases) beyond 12 months. HSK occurred at 5.67 ± 6.17 months after corneal transplantation.

### 3.4 HSK classification and intraocular pressure

Among the 40 cases (40 eyes) of patients with HSK after corneal transplantation, 11 cases (11 eyes) (27.5% of the 40 cases) had epithelial HSK, 8 cases (8 eyes) (20% of the 40 cases) had stromal necrotizing HSK, 17 cases (17 eyes) (42.5% of the 40 cases) had combined epithelial, stromal, and endothelial HSK, and 4 cases (4 eyes) (10% of the 40 cases) had endothelial HSK.

Three cases (3 eyes) (7.5% of the 40 cases) of stromal necrotizing HSK had elevated intraocular pressure, 1 case (1 eye) (2.5% of the 40 cases) of endothelial HSK had elevated intraocular pressure, 8 cases (8 eyes) (20% of the 40 cases) of combined epithelial, stromal, and endothelial HSK had elevated intraocular pressure, and 2 cases (2 eyes) (5% of the 40 cases) of epithelial HSK had elevated intraocular pressure.

### 3.5 Treatment outcomes

Among the 11 cases (11 eyes) of epithelial HSK, all achieved corneal transparency at 1-year follow-up after regular antiviral medication use. Among the 8 cases (8 eyes) of stromal necrotizing HSK, at 1-year follow-up, 2 cases (2 eyes) developed corneal nebula, 5 cases (5 eyes) developed corneal macula, and 1 case (1 eye) required repeat corneal transplantation due to near-perforation. Among the 17 cases (17 eyes) of combined epithelial, stromal, and endothelial HSK, at 1-year follow-up, 1 case (1 eye) developed corneal nebula, 4 cases (4 eyes) developed corneal macula, 10 cases (10 eyes) developed corneal leucoma, and 2 cases (2 eyes) required repeat corneal transplantation due to corneal perforation (see Table 1).

**Table 1:** Outcomes of non-endothelial HSK [cases (eyes)].

Infection type	Outcome					Total
	Corneal transparency	Corneal nebula	Corneal macula	Corneal leucoma	Corneal perforation	
Epithelial HSK	11 (11)	0	0	0	0	11 (11)
Stromal necrotizing HSK	0	2 (2)	5 (5)	0	1 (1)	8 (8)
Mixed epithelial, stromal, and endothelial HSK	0	1 (1)	4 (4)	10 (10)	2 (2)	17 (17)

Among the 4 patients (4 eyes) with endothelial HSK, outcomes at 1-year follow-up showed grade 0 corneal transparency in 2 patients (2 eyes) and grade 2 corneal transparency in 2 patients (2 eyes) (see Table 2).

**Table 2:** Treatment outcomes of endothelial HSK [cases (eyes)].

Type	Outcome			Total
	Grade 0 corneal transparency	Grade 1 corneal transparency	Grade 2 corneal transparency	
Endothelial HSK	2 (2)	0	2 (2)	4 (4)

### 3.6 Special case analysis

**Figure 2:**
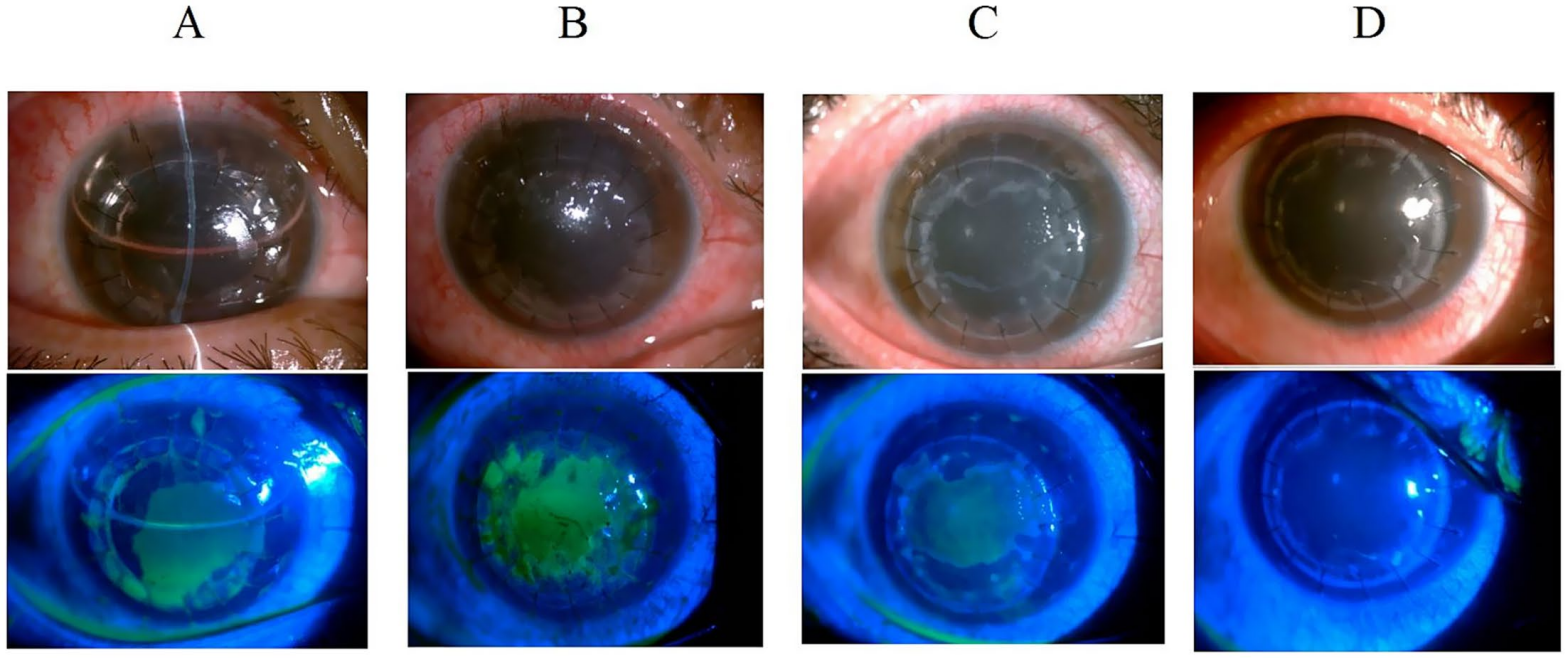
Slit-lamp examination of a 24-year-old male with keratoconus following deep anterior lamellar keratoplasty. **(A)** Graft clarity at 3 days post-operation with anterior chamber air bubbles and epithelial defects. **(B)** Persistent epithelial defect at 1 month with corneal edema and decreased transparency despite bandage contact lens and steroid therapy. **(C)** Continued graft transparency decline at 2 months with persistent epithelial defects, prompting systemic antiviral treatment. **(D)** Corneal clarity restoration 1 month after antiviral therapy initiation, with negative fluorescein staining.

**Figure 3:**
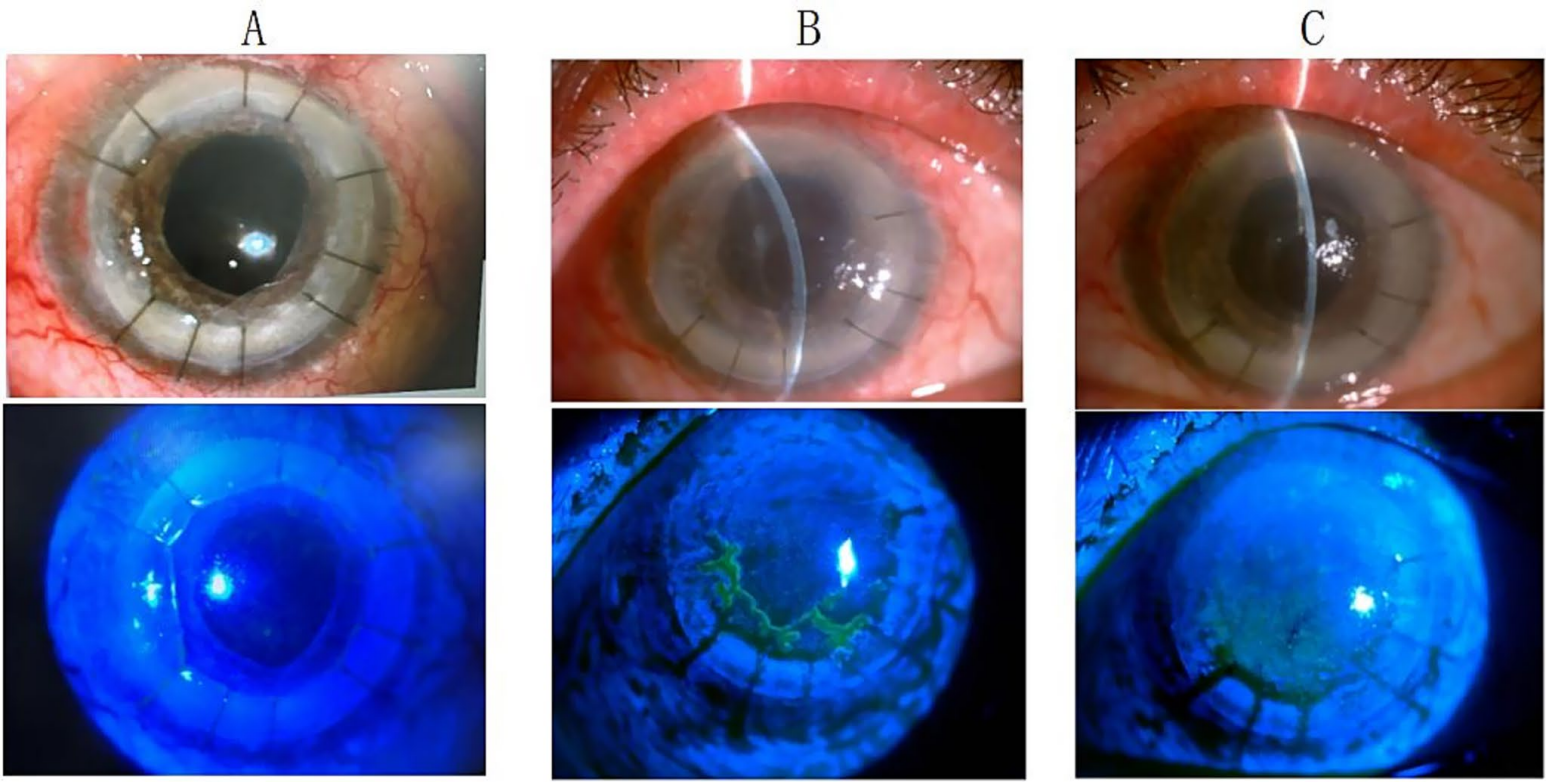
Anterior segment findings in a 49-year-old male following second penetrating keratoplasty. **(A)** Clear corneal graft with negative fluorescein staining at 3 months post-transplantation. **(B)** Corneal edema with dendritic epithelial defect positive for fluorescein staining at 4.5 months post-transplantation. **(C)** Improved corneal edema with negative fluorescein staining after 2 weeks of oral antiviral therapy.

We present two representative cases highlighting the importance of considering HSK when graft transparency declines after keratoplasty. The first case involved a post-DALK patient with progressive graft deterioration and persistent epithelial defects that showed limited response to conventional therapy. Clinical suspicion of HSK prompted systemic antiviral treatment, leading to significant improvement in corneal clarity and epithelial healing. The second case demonstrated HSK development in a post-PKP patient, presenting with graft edema and characteristic dendritic epithelial defects typical of viral keratitis. Prompt antiviral therapy resulted in notable improvement in both graft transparency and epithelial integrity. These cases suggest that HSK should be considered in the differential diagnosis when unexplained graft deterioration occurs, and that timely antiviral intervention may contribute to favorable clinical outcomes and graft preservation (Figures 2, 3).

## 4 Discussion

Corneal transplantation remains the only effective treatment for blindness caused by various corneal diseases. Previously, immune rejection and graft endothelial cell loss were considered the primary causes of graft decompensation following corneal transplantation. However, in recent years, an increasing number of studies have reported viral detection in grafts or aqueous humor from patients with graft decompensation (failure) after corneal transplantation, with viral presence detected in 3.9–14.3% of cases (3, 4).

In this study, the incidence of HSK after corneal transplantation was 9.73%. Some studies have reported that the incidence of HSK following corneal transplantation is higher than previously reported, with newly acquired HSV keratitis occurring in up to 71% of cases (10 patients) within 1 year after corneal transplantation (5, 6). Among the cases of graft ulceration occurring 2 years after penetrating keratoplasty, HSK patients can account for up to 50% (7).

In this study, among patients with pre-operative HSK, the postoperative infection rate was 14.77%. To date, the causes of postoperative HSK following corneal transplantation may include the following two mechanisms: First, viral infection resulting from donor corneal virus carriage. Studies have shown that the overall prevalence of human herpes virus DNA in donor corneal tissue is 8.24% (5). Remeijer et al. provided conclusive evidence (8) demonstrating HSV-1 transmission through penetrating keratoplasty. Case reports have even documented (9) that transmission of corneal tissue from HSV-1 seropositive donors to seronegative recipients may be particularly hazardous, as the virus may cause potentially blinding ocular infections in patients receiving immunosuppressive therapy, with severe cases potentially leading to HSK of other organs resulting in death. To prevent transmission of herpes simplex virus type 1 (HSV-1) from HSV-1 positive donors to HSV-1 naive recipients, serological matching between donors and recipients can be performed. If HSV-seronegative patients urgently require penetrating keratoplasty (PKP) and no seronegative donors are available, prophylactic use of acyclovir should be considered, particularly during immunosuppressive therapy (8). However, the risk of viral transmission from donor grafts to recipients remains difficult to eliminate, even with viral screening of donors. On one hand, the viral infection rate among donors is extremely low (<1%), making universal viral screening of all donors cost-prohibitive. On the other hand, due to the heterogeneous distribution of the virus within the cornea, viral detection results from the donor corneoscleral rim do not fully reflect the viral infection status of the actual graft tissue. Therefore, prevention and management of graft-related viral infections still rely heavily on regular postoperative follow-up examinations after corneal transplantation (3). Second, HSV can remain latent in the trigeminal ganglion (10). Patients undergoing corneal transplantation may have subclinical viral infections preoperatively, with the diseased cornea showing only nonspecific manifestations such as corneal edema or corneal opacity resulting from previous HSK. Studies have demonstrated that compromised immune status or prolonged topical application of corticosteroids and immunosuppressive agents can trigger reactivation of latent virus, which then travels retrogradely along neural axons to the ocular surface and corneal tissue, leading to secondary infection (11, 12). Therefore, frequent instillation of topical corticosteroid eye drops should be used cautiously following corneal transplantation. However, reducing the use of corticosteroids or immunosuppressive medications in all patients to prevent graft viral infection is not practically feasible.

The diagnosis of HSK following corneal transplantation is challenging, with most patients being misdiagnosed, leading to graft failure or decreased donor corneal transparency. This study implemented corresponding classification-based treatment protocols for different types of HSV keratitis (HSK) occurring after corneal transplantation. Dynamic transformation between different HSK types is possible, with type conversion or secondary infection occurring due to disease progression, changes in immune status, or external factors. Epithelial defects are commonly observed after corneal transplantation, and HSV-induced epithelial defects must be differentiated from those caused by other etiologies. A persistent epithelial defect in the donor cornea after keratoplasty poses a serious threat to graft survival. Studies have shown that 14–100% of patients develop early epithelial defects after penetrating keratoplasty (PK), which typically heal within 1 week postoperatively (13). However, 3–7% of patients remain unhealed after 2 weeks, presenting with persistent epithelial defects. Male sex, increased age, graft diameter >9 mm, bacterial and viral keratitis, and systemic diseases were independent risk factors for postoperative epithelial defects (13). Additionally, poor corneal neurotrophic status, aqueous tear-deficient dry eye syndrome, infrequent blinking, phthisis bulbi, reduced corneal sensitivity, impaired tear secretion (14, 15), postoperative graft rejection, graft infection, and limbal stem cell deficiency can also lead to persistent epithelial defects following corneal transplantation. In this study, among patients with HSV infection-induced epithelial defects following corneal transplantation, the mean age was 50 years, approximately 50% were male, and all donor graft diameters were <9 mm. For patients with epithelial defects due to ocular surface disorders, a strict topical medication regimen combined with bandage contact lens application or eye patching represents an effective treatment approach. In this study, all patients with post-transplantation epithelial defects underwent bandage contact lens application or eye patching, but there was no significant improvement. However, marked improvement was observed following systemic antiviral therapy, ruling out epithelial defects caused by poor ocular surface conditions in our patient cohort. Literature reports indicate that epithelial defects resulting from immunologic rejection primarily manifest as epithelial rejection lines, corneal edema, and opacity (13–16). Corticosteroids remain the first-line therapy for post-keratoplasty rejection and represent the gold standard for treating corneal transplant rejection (16, 17). Following corticosteroid treatment, rejection episodes typically resolve rapidly. In this study, when patients initially developed corneal epithelial defects or graft edema suspected to be rejection-related, increasing the corticosteroid dosage yielded no significant improvement. However, marked improvement was observed after initiating systemic antiviral therapy. In HSK, epithelial disease initially presents as small, punctate keratopathy, which subsequently coalesces into larger branching dendritic keratitis and may progress to extensive geographic ulceration. In this study, all patients with post-transplantation HSK received systemic antiviral therapy; for patients with epithelial HSK, the frequency and intensity of corticosteroid use were reduced, resulting in therapeutic improvement in all cases. Figure 3 demonstrates the dendritic epithelial staining pattern. Other studies have reported that new-onset HSK after transplantation can occur in both the graft and recipient tissues, or even throughout the entire corneal graft and the surrounding host bed. *In vivo* confocal microscopy examination reveals dendritic cell accumulation at the junction between diseased and clear corneal areas, with extensive dendritic cell infiltration present only in regions overlying epithelial or endothelial rejection lines (18–20). In this study, HSK epithelial infections predominantly involved the recipient bed, and bacterial, fungal, and Acanthamoeba infections were clinically excluded in all patients with epithelial defects. All patients undergoing corneal transplantation had intact limbal stem cells preserved.

Furthermore, endothelial HSK must be differentiated from endothelial rejection, as both conditions share numerous clinical similarities, making clinical differentiation challenging. The preoperative primary disease provides an important reference value for distinguishing between these two conditions. Elevated intraocular pressure predominantly occurs in patients with endothelial HSK, with keratic precipitates (KPs) typically presenting in a linear pattern that crosses the donor–recipient junction, accompanied by stromal edema in both donor and recipient corneas. Endothelial HSK often responds poorly to corticosteroid monotherapy, but demonstrates significant improvement following the addition of antiviral agents. In contrast, endothelial rejection responds markedly to corticosteroid treatment (28, 29). Shimomura and Higaki (21) observed that KPs in endothelial rejection are predominantly located on the posterior graft surface, with fewer occurring on the recipient bed. The KPs appear as a linearly oriented wave that originates at the periphery of the graft and progresses towards its center of the graft. The border of the corneal edema is characterized by this line (22). In this study, postoperative elevated intraocular pressure was frequently associated with endothelial HSK, and HSV infection-related graft stromal edema consistently involved the recipient bed. Additionally, KPs were often obscured due to corneal edema in our cohort. One case (one eye) presented with endothelial HSK viral infection following corneal transplantation. During follow-up examination, KPs were observed crossing the donor–recipient junction with stromal edema present in both donor and recipient corneas, subsequently developing concurrent epithelial HSK. The diagnosis of viral endotheliitis is challenging and requires an accurate diagnosis based on clinical features or etiological examination. When immunosuppressive therapy alone yields suboptimal results, therapeutic diagnosis can be attempted by administering systemic antiviral therapy to control the condition and maximize graft function preservation (18). In this study, one case (one eye) developed corneal edema initially suspected to be rejection-related. Increasing the frequency and dosage of topical or systemic corticosteroids resulted in worsening graft edema. Subsequently, reducing the frequency and dosage of topical corticosteroids while continuing oral systemic antiviral therapy led to marked improvement.

In this study, patients with endothelial or necrotizing stromal HSK without etiological diagnosis who showed clinical improvement after acyclovir or valacyclovir treatment were also considered to have HSV infection. Given that other viral infections (such as cytomegalovirus or other human herpesvirus infections) typically do not respond significantly to acyclovir or valacyclovir treatment, patients lacking etiological evidence who demonstrated clinical improvement following acyclovir or valacyclovir therapy were all classified as HSK.

To date, corticosteroid dosing following corneal transplantation for HSK remains one of the challenging issues faced by ophthalmologists. Oral antiviral medications can reduce the risk of herpetic keratitis recurrence in eyes that have undergone corneal transplantation within the first 12 months (23, 31, 32). Herpetic infection and clinical immunologic rejection may occur simultaneously and act synergistically (26, 27, 30). For patients whose preoperative primary disease was HSK, studies have also demonstrated that patients undergoing penetrating keratoplasty (PKP) for sequelae of herpes simplex virus (HSV) keratitis are at higher risk for adverse corneal allograft outcomes when compared to individuals undergoing grafting for conditions such as keratoconus and Fuchs’ corneal dystrophy (24). The postoperative course can be complicated by high rates of HSV recurrence, graft rejection, and graft failure. Topical corticosteroids can reduce the risk of immunologic rejection; however, corticosteroid use may precipitate HSK and even lead to corneal melting or perforation. In this study, patients with post-transplantation HSK should receive aggressive systemic antiviral therapy, with systemic and topical corticosteroids administered judiciously based on the epithelial status, infection severity, and rejection activity. Corticosteroid therapy requires careful titration to achieve adequate anti-rejection efficacy while avoiding disease exacerbation.

Studies have reported that patients with a history of HSV keratitis have a high risk of donor graft failure due to immune mechanisms (19). Therefore, for patients with a history of HSV keratitis, antiviral therapy should be administered preoperatively and for at least 1 year postoperatively (12, 20). Oral antiviral medications can reduce the risk of herpetic keratitis recurrence in eyes that have undergone corneal transplantation within the first 12 months (25). In this study, all patients with etiologically confirmed or highly suspected HSK received systemic antiviral therapy perioperatively, yet these patients could still experience HSK episodes. For patients in the acute infection phase, HSV remained active at the time of surgery, and postoperative corticosteroid administration further promoted HSV activation, resulting in persistent HSK following corneal transplantation that was difficult to resolve, with high susceptibility to recurrence even after HSV infection was controlled. For patients with HSK leucoma, postoperative corticosteroid use and factors such as decreased host resistance could induce HSK recurrence. For grafts with full-thickness HSK that eventually developed irreversible opacity, we consider the following reasons: (1) Patients did not follow up on time or did not use systemic antiviral medications as prescribed. (2) Patients had HSK or corneal leucoma preoperatively, and even with aggressive use of systemic antiviral medications postoperatively, improvement was very slow, ultimately leading to an inevitable decline in graft transparency. This demonstrates the severe harm that HSV causes to corneal grafts. Some scholars also believe that antiviral medications should be used routinely regardless of whether the primary disease is herpes simplex viral keratitis or not. We will further refine and strengthen prophylactic antiviral treatment strategies following corneal transplantation in future clinical practice.

For cases with severe inflammatory response, large and deep ulceration, and persistent epithelial defects, studies have reported that pharmacological therapy combined with amniotic membrane transplantation can be beneficial (7). Amniotic membrane tissue promotes epithelial healing, reduces inflammatory response, and inhibits fibroblast proliferation and neovascularization.

Some advocates propose routine HSV testing of all donor corneas regardless of clinical suspicion, to detect, treat, and prevent potential recurrent herpetic infections in corneal grafts, thereby supporting graft survival. However, this aggressive diagnostic approach is not commonly employed due to cost considerations and constraints in the medicolegal environment. Even in patients with a history of herpetic keratitis (HK), low viral DNA loads may result in false-negative biopsy results (12).

## 5 Conclusion

The period of 1–3 months following corneal transplantation is a peak time for HSK. Regular follow-up is essential for all patients’ post-surgery. For those with a history of HSK, vigilance for HSV recurrence is critical, while timely diagnosis and differentiation of HSK are crucial for non-HSK patients. When administering systemic antiviral therapy, it is important to adjust the frequency and intensity of steroid treatments promptly to prevent irreversible graft opacity.

## Data Availability

The original contributions presented in the study are included in the article/supplementary material, further inquiries can be directed to the corresponding authors.
