# Correction: Clinical manifestations and outcomes of HSK following corneal transplantation

**DOI:** 10.3389/fmed.2026.1809061

**Published:** 2026-03-13

**Authors:** Dan Wu, Hui Huang, Min Zheng, Qi Chen, Zhou Zhou, Li Jiang, Yuanhua Li, Yiyun Liu, Baikai Ma, Guoqing Chen, Yujun Huang, Fengping Cen, Binghua Chen, Fengmei Li, Hong Qi, Fan Xu, Qianqian Lan

**Affiliations:** 1 Department of Ophthalmology, The People's Hospital of Guangxi Zhuang Autonomous Region & Guangxi Key Laboratory of Eye Health & Guangxi Health Commission Key Laboratory of Ophthalmology and Related Systemic Diseases Artificial Intelligence Screening Technology & Institute of Ophthalmic Diseases, Guangxi Academy of Medical Sciences, Nanning, China; 2 Department of Ophthalmology, Peking University Third Hospital & Beijing key Laboratory of Restoration of Damaged Ocular Nerve, Peking University Third Hospital, Beijing, China

**Keywords:** herpes simplex keratitis, corneal transplantation, new-onset HSK, recurrent HSK, HSK classification

The article type of this article was erroneously defined as “Review”. The correct article type is “Original Research.”

Accordingly, the ethics and data availability statements required for this article type have been added and appear below.

## Data Availability

The original contributions presented in the study are included in the article/supplementary material, further inquiries can be directed to the corresponding authors.

